# Mechanisms and Prediction of Ischemic Stroke in Atrial Fibrillation Patients

**DOI:** 10.3390/jcm12206491

**Published:** 2023-10-12

**Authors:** Errol Aarnink, Maxime Zabern, Lucas Boersma, Michael Glikson

**Affiliations:** 1 Department of Cardiology, St. Antonius Hospital, 3435 CM Nieuwegein, The Netherlands; 2 Jesselson Integrated Heart Center, Shaare Zedek Medical Center, Jerusalem 9103102, Israel; 3 Department of Cardiology, Amsterdam University Medical Center, 1105 AZ Amsterdam, The Netherlands

**Keywords:** atrial fibrillation, stroke prevention, stroke prediction, left atrial appendage

## Abstract

Atrial fibrillation (AF) is the most common arrhythmia in adults worldwide and represents an important burden for patients, physicians, and healthcare systems. AF is associated with substantial mortality and morbidity, due to the disease itself and its specific complications, such as the increased risk of stroke and thromboembolic events associated with AF. The temporal relation between AF episodes and stroke is nonetheless incompletely understood. The factors associated with an increased thromboembolic risk remain unclear, as well as the stroke risk stratification. Therefore, in this review, we intend to expose the mechanisms and physiopathology leading to intracardiac thrombus formation and stroke in AF patients, together with the evidence supporting the causal hypothesis. We also expose the risk factors associated with increased risk of stroke, the current different risk stratification tools as well as future prospects for improving this risk stratification.

## 1. Introduction

The worldwide burden of ischemic stroke is significant, with almost eight million cases on an annual basis, resulting in nearly 64 million disability-adjusted life years. More than three million people die from ischemic stroke per year. Importantly, the incidence of ischemic stroke has increased the last decades [1], making its etiology an increasingly relevant topic.

**Figure 1:**
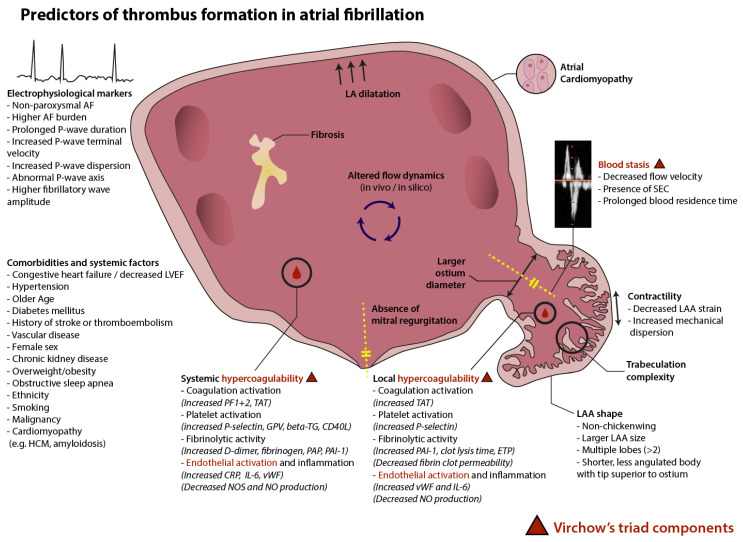
Predictors of thrombus formation in atrial fibrillation AF: atrial fibrillation, LVEF: left ventricular ejection fraction, HCM: hypertrophic cardiomyopathy, PF1 + 2: prothrombin fragment 1 + 2, TAT: thrombin-antithrombin complex, GPV: glycoprotein V, beta-TG: beta-thromboglobulin, CD40L: CD40 ligand, PAP: plasmin-alfa2-antiplasmin complex, PAI-1: plasminogen activator inhibitor, CRP: C-reactive protein, IL-6: interleukin-6, vWF: von Willebrand Factor, NOS: nitric oxide synthase, NO: nitric oxide, ETP: endogenous thrombin potential, SEC: spontaneous echocardiographic contrast, LAA: left atrial appendage.

Although the cause of ischemic stroke can be multifactorial, the presence of atrial fibrillation (AF) brings forth a fivefold increase in ischemic stroke risk [2]. Altered interatrial flow dynamics in AF, among other processes, predispose patients to thrombus formation. Embolization of a thrombus from the left atrium (LA) in the systemic circulation can lead to ischemic stroke when the migrated thrombus blocks a cerebral artery. The long-term mortality rate after cardioembolic stroke is highest among ischemic stroke subtypes [3]. Especially the occurrence of large artery cortical stroke is common in the presence of AF [4]. The possible subsequent disruption of higher cognitive functions has a substantial impact on quality of life [5]. It is therefore of paramount importance to better understand the mechanisms leading to ischemic stroke in patients with AF. In the current review, we aim to establish risk factors for ischemic stroke in AF patients and contemplate their role in prediction within this high-risk population (Figure 1).

## 2. Left Atrial Properties and Ischemic Stroke

### 2.1. Migration of Cardiac Thrombi

Thrombus formation can occur in any heart chamber. Right-sided thrombi may migrate to the pulmonary circulation, causing pulmonary embolism. Thrombi causing pulmonary embolism nonetheless only rarely originates in the right atrium or ventricle itself; more often, emboli arise from deep venous thrombosis in the lower-extremity veins [6]. Although smaller thrombi may induce pulmonary infarction or pleuritis, they infrequently cause clinically significant impairment of gas exchange or compromised hemodynamics. 

Left-sided thrombi may have more substantial implications in the case of migration, even when limited in size. Subsequent small cerebral infarction can result in significant cognitive disabilities depending on the location of the lesion [7]. When these disabilities endure, they significantly impact quality of life. Therefore, unraveling the left atrial properties contributing to ischemic stroke is of vital importance for understanding the etiology of cardioembolic stroke. 

### 2.2. Virchow’s Triad in Atrial Fibrillation

In general, thrombus formation is predisposed due to the presence of one or more components of Virchow’s triad, including endothelial activation, altered blood flow, and hypercoagulability. This concept is commonly associated with the occurrence of deep venous thrombosis, but can be applied to the development of left atrial thrombus as well, especially in the context of AF [8]. 

The poor atrial contraction resulting from AF leads to the stasis of blood in the left atrium and left atrial appendage (LAA), altering local flow conditions. Slow left atrial blood flow can result in a sludge-like structure visible as spontaneous echocardiographic contrast (SEC), possibly due to the formation of platelet–monocyte aggregates [9]. SEC is associated with several factors, including a reduced cardiac index, a larger left atrial diameter, and permanent AF. Importantly, this phenomenon is also associated with prior thromboembolism and can develop into a thrombus [10]. 

Various biomarkers of endothelial and coagulation activation, inflammation, and fibrinolysis are elevated in AF patients. The impairment of flow dynamics predisposes patients to coagulation activation, which leads to increased serum levels of coagulation activation markers. Prothrombin fragment 1 + 2 (PF1 + 2) is formed after the generation of thrombin. The binding of thrombin results in a thrombin–antithrombin complex (TAT). PF1 + 2 and TAT therefore give an indication of thrombin generation. Both markers are elevated in patients with AF [11,12,13]. This is accompanied by an increase in downstream markers for fibrinolytic activity. Fibrin degradation leads to the formation of D-dimer. D-dimer levels also are elevated in patients suffering from AF and a higher D-dimer level is associated with the occurrence of stroke or systemic embolism [14,15]. Moreover, a low LAA flow velocity (<30 cm/s) and SEC may be associated with higher systemic D-dimer levels [16]. Other markers of fibrinolytic activity, such as plasmin-alpha2-antiplasmin (PAP) complex, plasminogen activator inhibitor (PAI-1), fibrinogen, and tissue plasminogen activator (TPA), could also have a role in thromboembolic prediction in AF patients [17,18,19,20]. The plasma fibrin clots found in AF are very compact, which can be expressed as reduced fibrin clot permeability. This complicates the process of fibrinolysis and increases ischemic stroke risk [21].

Although coagulation activation likely is the predominant process for thrombus formation in AF, the activation of platelets also plays a role. Activated platelets release several proteins that can be used as surrogate markers for in vivo platelet activation. Multiple soluble platelet activation biomarkers show an increase in patients with AF, such as P-selectin, glycoprotein V, beta-thromboglobulin, and CD40 ligand [22]. 

Finally, endothelial activation and inflammation play a role in the predisposition to thrombus formation in AF. The low local blood flow conditions downregulate the endothelial expression of nitric oxide synthase (NOS) and nitric oxide (NO). These markers play an important role in the regulation of vascular tone and have significant antithrombotic effects. AF leads to a reduced production of NO and NOS. Interestingly, PAI-1 is regulated by NO, which may explain increased levels of this fibrinolytic marker in AF [23]. Furthermore, the endothelial function can be assessed by measuring plasma’s von Willebrand factor (vWF). This marker shows an increase in AF patients, with higher levels predicting stroke—but only in univariate analysis [24]. The relation of vWF with stroke therefore remains unclear. Furthermore, inflammatory markers such as C-reactive protein (CRP) and interleukin-6 (IL-6) are increased in AF [25]. Over time, inflammatory processes result in fibrosis and atrial remodeling [26].

These findings on the activation of coagulation, platelets, fibrinolysis, and the endothelium suggest that AF influences multiple processes involved in thrombus formation, propagation, and maintenance. AF patients therefore demonstrate an increased thrombotic tendency, a phenomenon often described as a prothrombotic state [27]. 

### 2.3. Atrial Remodeling, Fibrosis, and Atrial Fibrillation

AF leads to contractile, structural, and electrophysiological changes over time [28]. Furthermore, the presence of AF by itself leads to higher susceptibility to subsequent arrhythmic episodes [29]. AF therefore has a natural disease progression, worsening from paroxysmal to persistent and then permanent, with the success rate of cardioversion gradually declining. The presence of AF is correlated with left atrial dilatation, with a prolonged duration of AF as a predictor of a larger LA diameter [30,31]. Although not consistently present in the literature [32], LA enlargement may predispose patients to thrombus formation and stroke [33,34]. For AF patients, this may add to the increased stroke risk over time due to disease progression. This is accompanied by the association of an LAA thrombus with an AF type (paroxysmal, persistent, or permanent) [32]. However, AF duration and burden alone fail to fully explain the cardioembolic risk, and the temporal relationship between embolic events and AF episodes is unclear [35]. 

Interestingly, atrial remodeling may already start before clinical AF appears. In the PREDICT-AF trial [36], the LAA was excluded and blood was collected in patients without AF undergoing cardiothoracic surgery. Patients that developed AF during a 2-year follow-up period (12%) showed increased levels of multiple plasma and tissue biomarkers, combined with altered gene expression. This implies that atrial remodeling precedes AF and that the arrhythmogenic substrate can be detected at an early stage.

Remodeling due to AF is accompanied by the formation of fibrosis in the LA. Fibrotic tissue has poor conductive properties, which may lead to the progression of AF due to reentry mechanisms. Left atrial fibrosis can be quantified using (delayed enhancement) magnetic resonance imaging. Patients with a prior history of stroke had a significantly higher percentage of LA fibrosis in a study carried out by Daccarett et al. In multivariate logistic regression analysis, LA fibrosis independently predicted stroke and more severe fibrosis was associated with higher odds for stroke [37]. In addition to stroke, LA fibrosis also correlates with the presence of SEC or an LAA thrombus [38], underlining its importance in disease progression and thrombosis.

Remodeling and fibrosis influence electrophysiological function, yielding electrocardiographic surrogate markers for these underlying processes that can be more easily assessed than when performing (magnetic resonance) imaging. During AF, novel electrocardiographic methods can be used to precisely determine the fibrillatory wave amplitude and dominant frequency, providing an idea of AF complexity. Retaining sinus rhythm after catheter ablation and electrocardioversion shows a relation with these markers [39,40]. In sinus rhythm, the mean P-wave duration and terminal velocity in V1 (as an indicator of left atrial excitation) may be influenced by remodeling, thus providing an early marker for AF propensity [41]. Both P-wave duration and terminal velocity, along with P-wave dispersion (the time between the longest and shortest P-wave duration within different leads), have been associated with thrombus formation within the LAA [42]. Maheshwari et al. [43], in their study on almost 3000 patients, presumed that the P-wave morphology in sinus rhythm presented an opportunity to detect pro-thrombotic atrial remodeling via measurement of the P-wave indices. They observed that an abnormal P-wave axis was associated with an increased risk of stroke independent of the CHA2DS2-VASc variables. They thus developed the P2-CHA2DS2-VASc score, which improves the ischemic stroke prediction compared to the regular CHA2DS2-VASc score. Similarly, fibrillatory wave amplitude may be associated with LAA dysfunction and thromboembolism [44], but the literature on its relation to stroke is scarce. These markers may provide an estimation of the severity of electrophysiological remodeling within AF and thereby stroke propensity. 

### 2.4. Atrial Cardiomyopathy

Historically, AF has been described as the main driving factor contributing to cardioembolic stroke, with blood pooling in the atria during irregular contractions as the main cause of thrombus formation. However, some inconsistencies remain with this theory. Following this rationale, the duration of AF episodes and thus the type of AF (paroxysmal, persistent, or permanent) should be an important predictor of ischemic stroke. Although the thromboembolic risk seems slightly higher in non-paroxysmal AF [45], a strong relation between the presence of (sub-clinical) AF and the occurrence of ischemic stroke is uncertain [46]. Moreover, the temporal relationship between an episode of AF and the occurrence of a thromboembolic event is not so clear-cut [35]. Even after successful ablation for AF, the guidelines recommend the continuation of oral anticoagulation therapy (OAT) in those at increased thromboembolic risk [47]. Time in AF may therefore insufficiently predict ischemic stroke risk.

The attention on atrial cardiomyopathy (ACM) as a risk factor for ischemic stroke has grown in the past decade. ACM is defined as “any complex of structural, architectural, contractile or electrophysiological changes affecting the atria with the potential to produce clinically-relevant manifestations” [48]. The occurrence of clinical AF may be the manifestation of longer-progressing ACM. It is likely that ischemic stroke is a consequence of the interplay between AF, ACM, and local and systemic risk factors. Patients without clinical AF, but demonstrating signs of ACM combined with systemic risk factors, might benefit from anticoagulant use.

The ongoing ARCADIA trial [49] investigates a population of patients with a history of embolic stroke of undetermined source (ESUS) and the characteristics of ACM. To be eligible for the study, patients should have at least one of the following markers: a P-wave terminal force >5000 µV × ms in lead V1 on an electrocardiogram, serum NT-proBNP > 250 pg/mL, and/or a left atrial diameter index ≥ 3 cm/m^2^ on an echocardiogram. In this randomized, double-blind trial, the use of apixaban is compared to aspirin for the primary efficacy outcome of recurrent stroke of any type. Importantly, patients with known AF are excluded from this trial. ARCADIA may further elucidate the importance of ACM as a substrate for ischemic stroke in the absence of AF and provide insights into the optimal approach to minimizing stroke risk. Of note, the NAVIGATE-ESUS trial [50] investigated rivaroxaban versus aspirin in a much broader population with ESUS in the absence of identified risk factors for a cardiac source of embolism. This trial was ended prematurely due to higher bleeding rates in the rivaroxaban group, without evidence for improved efficacy. Likewise, dabigatran did not result in a reduced recurrent stroke rate compared to aspirin in the RE-SPECT ESUS trial [51], although not at the cost of higher bleeding risk. The future will point out whether the subpopulation with characteristics of ACM will benefit from OAT.

## 3. The Left Atrial Appendage as the Cradle of Thrombi

In the etiology of cardioembolic stroke in patients suffering from AF, the LAA is a structure of particular interest. Although the LAA plays a role in homeostasis, hemodynamics, and arrhythmogenesis [52], it is mostly infamous for its role in thrombus formation. In their 1996 review, Blackshear and Odell reported that 91% of LA thrombi in patients with non-rheumatic AF originated in the LAA [53]. This observation paved the way for occlusion of the LAA as a method for mechanical protection from ischemic stroke and systemic embolism. But why may thrombi so often develop in the LAA?

### 3.1. Morphologic Features

The shape and structure of the LAA vary widely among individuals, and a person’s LAA may be as unique as their fingerprint. Nevertheless, an attempt has been made to classify the various LAA shapes into some creatively devised main categories. The most common LAA morphology is the chicken wing, present in about half of patients. Other LAA shapes include the windsock, cactus, and cauliflower, among a plethora of others. These morphologic types are of clinical importance, as chicken wing morphology is associated with a lower rate of stroke/TIA compared to other LAA types [54,55], although the interobserver reproducibility of classifying the shape of the LAA is poor and remains one of the limitations of the LAA shape criteria [56]. In addition to the general shape, other morphologic features may also be of importance for the development of LAA thrombi. During postmortem examination, Ernst et al. found appendages containing thrombi to be significantly larger than those without thrombi [57]. The importance of LAA size as a contributor to thromboembolic risk was confirmed by Taina et al., showing larger LAA volume (adjusted for body surface area) in patients with cryptogenic stroke compared to age- and gender-matched controls [58]. 

While several anatomical terms exist for the classification of the outer shape of the LAA, the inside is hard to classify qualitatively due to its complex structure and numerous trabeculations. For this purpose, quantitative assessment methods have been developed. The mathematical concept of fractal dimension can be used to quantify trabeculation complexity, which showed an association with history of stroke [59]. This finding is in line with earlier observations that more complex appendages are prone to developing thrombi, especially when consisting of more than two lobes [34] or having more extensive trabeculations [60]. In addition to this method, statistical shape analysis can quantify the shape of the endocardial LA/LAA surface using automated computation of landmark points on computed tomography (CT) or magnetic resonance imaging (MRI). In a study by Bieging et al. [61], AF patients with prior stroke showed different LAA morphology. High-risk LAAs had a broader, shorter, and less angulated body with the tip superior to the ostium, whereas low-risk LAAs were longer and more angulated. Interestingly, endocardial LA morphology was not of importance. 

Furthermore, the contractility of the LAA affects the likelihood of local thrombus development, irrespective of LA contractility. Strain is an echocardiographic biomarker of myocardial tissue deformation during systole and thus gives an indication of local myocardial contractility and mechanistic function [62]. When measured in the LAA, the strain parameters are significantly lower in AF patients with thrombi or SEC compared to AF patients without [63,64]. LAA strain proved higher in patients with sinus rhythm and lower in patients with permanent AF as opposed to patients with paroxysmal AF [65]. Additionally, strain echocardiography can assess the timing of LAA contractions, quantifying contractile irregularity as mechanical dispersion. Increased mechanical dispersion has been demonstrated to correlate with the occurrence of LAA thrombi or SEC [66]. In a study by Mao et al. [67], patients with a prior thromboembolic event had significantly lower LAA strain in all categorized LAA regions (basal, middle, apical, and global longitudinal strain) as well as higher mechanical dispersion, with only mechanical dispersion remaining statistically significant in multivariate logistic regression analysis. Further research will have to show whether LAA strain measurements can play a role in thromboembolic risk stratification.

### 3.2. Flow Dynamics

LAA shape, complexity, and contractility all contribute to local hemodynamics and (the stagnation of) flow. Maximum flow velocity, measured just below the LAA ostium, is an important marker of local thrombogenicity. It is associated with SEC and LAA thrombi as well as with cardioembolic stroke [68,69]. Interestingly, known predictors of thromboembolic risk, such as LA size, left ventricular ejection fraction, type of AF, age, and sex, all influence LAA flow velocity [70]. Moreover, LAA flow velocity negatively correlates with the LAA orifice area [71]. A larger orifice area has been linked to thrombus formation and ischemic stroke irrespective of LAA volume [60,72,73] and may be one of the causes of low flow velocity. Interestingly, the orifice area is smaller in chicken wing appendages [74], possibly contributing to the lower stroke risk in this morphology type. 

LA and LAA flow patterns are influenced by the presence of mitral regurgitation, a phenomenon that has been hypothesized to have a protective effect on stroke risk in AF patients, especially in the context of LA enlargement [75,76]. However, newer studies in larger cohorts debate this effect, especially when accounting for modern-day risk scores such as CHA2DS2-VASc [77]. Also, left ventricular ejection fraction (LVEF) could indirectly influence LA/LAA flow dynamics. Although non-uniformly described in literature, it may be associated with the occurrence of LAA thrombi and ischemic stroke in AF [32,78,79,80].

The maximum LAA flow velocity is an important marker of local thrombogenicity, yet it is limited by its two-dimensional character. The flow velocity depends on the moment of measurement, both within the cardiac cycle and depending on the underlying rhythm—AF or sinus rhythm. Furthermore, it may vary between observers due to different measurement locations. Newer technologies could overcome these inconsistencies. Four-dimensional flow analysis is a new imaging modality that enables the quantification of complex intracardiac flow patterns using particle tracking. Demirkan et al. recently emphasized its potential in the context of left ventricular thrombus formation following myocardial infarction [81]. In their study using cardiac magnetic resonance imaging, patients with left ventricular thrombi demonstrated a reduced direct systolic flow and a more delayed and residual flow. Additionally, the flow patterns changed, with reduced vorticity (i.e., rotational motion) and kinetic energy in patients with myocardial infarction compared to healthy controls. These findings illustrate the potential of four-dimensional flow analysis in further understanding local flow patterns and their importance in thrombogenesis, especially when applied to the LA and LAA. In this regard, Markl et al. [82] showed that LA and LAA volume were positively correlated with four-dimensional-flow-analysis-derived LA and LAA velocities and negatively correlated with LA/LAA stasis. The LAA flow was slower than the LA flow in patients in sinus rhythm, but this did not hold true for patients in AF. The flow patterns in AF may therefore be more complex than can be expressed using only flow velocity. However, no analysis for vorticity or kinetic energy was performed in their study. Interestingly, first reports show that closing the LAA may improve the LA flow dynamics measured by four-dimensional magnetic resonance imaging [83]. Recently, LAA four-dimensional flow analysis has also been applied to cardiac CT, with similar outcomes to magnetic resonance imaging [84]. 

While four-dimensional flow analysis uses in vivo measurements, computational fluid dynamics (CFD) aim to simulate flow dynamics in silico. Using this method, blood residence time in the LA and LAA can be simulated. The residence time appears to be longer in patients with AF versus patients with patent foramen ovales, but also in patent ovale patients versus healthy subjects [85], suggesting its relevance to cardioembolic and cryptogenic stroke. Furthermore, CFD show that the LAA inflow mainly originates from the left pulmonary veins and that the position of the LAA with respect to the left pulmonary veins influences the LAA flow dynamics and possibly thrombogenicity [86,87]. In line with in vivo observations, the CFD-derived ostium diameter and LAA length potentially predict thrombogenic risk [88]. 

### 3.3. Local Prothrombotic Milieu

AF is associated with the activation of coagulation, platelets, and fibrinolysis. However, most research on hematological biomarkers in AF describes peripheral venipuncture for blood collection, thus assessing systemic biomarker levels. These values do not necessarily portray the local hemostatic status in the LA or LAA. The LAA is not only unique in terms of morphology and flow dynamics, but also has a very specific internal milieu, contributing to another pillar of Virchow’s triad of thrombosis. In the HEART-CLOT trial [89], the biomarkers of thrombin generation and fibrinolysis were measured in blood collected from six different locations, i.e., from the right atrium, right ventricle, left atrium, left ventricle, left atrial appendage, and peripheral venipuncture. The blood collected from the LAA showed reduced fibrin clot permeability, increased clot lysis time, and increased endogenous thrombin potential compared to the peripheral blood. Other studies also demonstrate increased local LAA markers compared to systemic markers in AF patients. The systemic effects of AF seem more distinct in the LAA, with indications of altered local markers of coagulation activation (TAT), platelet activation (P-selectin), endothelial activation (vWF and NO production), and fibrinolytic activity (PAI-1) [90,91,92,93]. These findings suggest a more thrombogenic milieu in the LAA than in the systemic circulation, confirming its particular importance for thrombus formation in AF. 

## 4. Temporal Association between AF and Stroke

Despite its worldwide burden and the evident association between AF and thromboembolic events (TEs), the temporal association between the onset of an episode of atrial arrythmia and the onset of an ischemic event remains unclear and controversial in the literature. 

In a recent clinical study on more than 7000 patients with a first diagnosis of stroke or AF, the authors found out that the temporal relationship between AF and stroke showed a clustering of diagnoses of both diseases within the years around the diagnosis of the other disease [94]. Moreover, the diagnosis of AF with a stroke or a stroke with AF is associated with increased mortality compared to the diagnosis of each of the diseases independently. This global association in diagnoses of the two diseases suggests a temporal correlation, but as we discussed earlier, AF can both be the cause of the stroke/TE or just a marker or risk. 

Aiming to answer that question and to prove the causality of an episode of AF in a patient presenting with stroke, some other studies looked at the temporal relationship between a stroke or TE and an episode of AF in a patient with cardiac implantable electronic devices (CIEDs). 

In their study, Daoud et al. [95] showed that, overall, 73% of the 40 patients presenting with stroke or a TE equipped with CIEDs did not present any episode of atrial arrythmia in the 30 days preceding the ischemic event. Moreover, in the 20 patients with documented AF or an atrial high-rate episode (AHRE) prior to the ischemic event, only 55% of them presented at least one episode in the 30 days preceding the event. Concordant results were observed by Brambatti et al. [35]. In their study, 36% of patients with CIED monitoring had an episode of AF or an AHRE > 6 min before the stroke, but only 8% presented an episode in the 30 days preceding the stroke. 

We can also notice that in those two studies, a number of patients (respectively, 15% and 15.6%) developed AF during CIED monitoring in the coming months after the index event of stroke or a TE, which could also suggest that the required elements to develop stroke, such as biological changes, atrial cardiomyopathy, and other clinical risk factors, were already met before the appearance of AF. In those cases, stroke preceded the appearance of AF.

In a more recent work on 891 patients, Singer et al. [96] evaluated in a crossover design study the incidental risk of stroke following an episode of AF detected by a CIED by comparing AF burden in the 30-day period prior to stroke to a control period (90–120 days before stroke), and thus evaluated the additional risk of stroke following an episode of atrial arrythmia. They showed that only 5.8% of patients had an informative pattern with an episode in the 30 days before the event of stroke without a previous episode in the control period. However, the risk of stroke in their study seems to be higher in the 1 to 5 days following an AF episode of 5.5 h or more, and even higher with an episode of AF duration of at least 23 h or more. A similar result was observed according to the same methodology by Turakhia et al. [97]. Table 1 summarizes the main studies on the temporal association between AF and stroke or TE.

Moreover, the question of the threshold of the minimal pro-embolic duration of an episode of AF or atrial burden also remains unknown. In the recent NOAH-AFNET 6 trial [98], no benefits were observed in the anticoagulation group versus placebo in term of the reduction of stroke in patients with implantable devices and atrial high-rate episodes of more than 6 min, but a higher risk of bleeding was present. 

## 5. Comorbidities and Vascular Risk Profile

On top of the anatomic and echocardiographic or CT risk factors discussed earlier, many other studies intended to identify the risk factors associated with an increased risk of stroke or thromboembolic events in order to stratify risk, identify high-risk patients, and eventually reduce risk with anticoagulation in those patients. 

Many studies have long shown that the main independent risk factors associated with an ischemic stroke or a thromboembolic event are a prior stroke or transient ischemic attack, ageing (moreover, an age > 75 years old), hypertension, diabetes, and the presence of structural heart disease (SHD) or heart failure (HF) [78,99].

More recent studies added a sex category (female) and the presence of vascular disease as independent risk factors, and the CHA2DS2-VASc score has been validated as more efficient in predicting the risk of stroke in patients with AF [100]. It is now routinely recommended to stratify the risk of stroke and indicate prophylactic anticoagulation [47]. 

But despite those clearly identified risk factors, some other clinical or biological parameters seem also to be associated with an increased risk of ischemic stroke or thromboembolic events, and might help to further identify patients with a significant risk of stroke in order to treat them.

Chronic kidney disease (CKD) is common in patients with AF (up to 15% of patients with AF meet the criteria for the diagnosis of CKD) and the prevalence of AF increases with the severity of renal function impairment [101]. CKD and a high level of blood creatinine have been associated in the literature with a higher risk of an ischemic stroke or a TE [102,103,104], and this risk seems to be even higher in patient with end-stage CKD. Treatment with VKA seems to reduce the risk of stroke and even the combined risk of stroke and bleeding in patients with AF with a high risk of stroke and CKD. A score, R2CHADS2, incorporating a reduced creatinine clearance into the classical CHADS2 has been proposed and evaluated, and offers a more precise prediction of stroke when compared to CHA2DS2-VASc or CHADS2 [105] in patients with native AF, but seems to have similar performance in predicting stroke in patients after AF ablation [106]. Chronic renal failure has also been included as a criterion in the ATRIA score [107] and also in the GARFIELD AF tool [108,109] which seems to have at least as good accuracy to predict stroke or TEs in patients with AF [108,110].

Ethnicity has also been identified as a specific risk factor. Whereas the risk of developing AF seems to be lower in non-white individuals, this population has a higher risk of stroke or TEs [111,112,113]. This might be due to a higher prevalence of cardiovascular risk factors but also to intrinsic or genetical factors. The higher risk of stroke is noted at baseline and might be due to the under-prescription of anticoagulants in this population, but this increased risk compared to the white population persists also in patients taking anticoagulants. Moreover, worse outcomes after stroke are also reported in non-white individuals. It is also interesting to note that the non-white population is often underrepresented in large trials on anticoagulation or left atrial appendage occlusion, and the results in this specific population might differ from the white population in term of the efficacity of treatment due to a different metabolism and a specific profile for complications. 

Being overweight (BMI 25 to 30 kg/m^2^) and especially obesity (BMI > 30 kg/m^2^) are associated with the development of AF, but they are also associated with a higher risk of ischemic stroke or TEs [114]. This increased risk remains statistically significant even after adjustment for classical embolic risk factors and cardiovascular comorbidities (CHADS2 and CHA2DS2-VASc) and may be modified by sex and BMI, as it seems to be even more important in obese individuals of male sex. 

Obstructive sleep apnea (OSA) is also a common finding associated with AF, and can also worsen the natural history of AF. Obstructive sleep apnea has also independently been associated with an increased risk of stroke in two different studies [115,116]. In their study, Yaranov et al. [115] found that this OSA-associated risk is even more significant in patients identified at “low risk” with an increased risk of stroke of 62% in patients with a CHA2DS2-VASc of 0 in patients with OSA compared to patients without. 

Some other clinical factors have been identified as being associated with a higher risk of stroke or TEs [101] such as smoking, malignancy, and some specific etiology of cardiomyopathy such as hypertrophic cardiomyopathy and amyloidosis. Left ventricular hypertrophy has been associated with increased stroke/TE risk, albeit in individuals without AF [117].

Some biological parameters have also been described as being associated with a higher risk of a thromboembolic event or stroke in AF patients, such as hypercoagulability evaluated using D-dimer [15] but also inflammatory markers such as CRP [118], as discussed in the first section. More specific biological markers as cardiac specific markers like troponin and NTpro-BNP were also associated with a greater risk of stroke [118,119]. This might be the reflection of an advanced state of ACM or HF or of a deterioration in the hemodynamic flow within the LA in patients with underlying SHD or persistent AF, but it also might be an independent marker of risk. Hyperlipidemia has also been independently associated with an increased risk of LAA thrombi in patients with AF undergoing transesophageal echocardiography (TEE) before cardioversion or ablation, with a correlation between the level of LDL-C and the risk of stroke. This increased risk was also observed in patients identified as “low-risk” patients according to CHA2DS2-VASc (≤1) [119]. 

Different attempts have been made to incorporate those new embolic risk factors into practical scores to evaluate the risk of stroke in a patient, resulting most of the time in an improved statistical performance, but most of those attempts consisted of simply adding one or two parameters to preexisting scores. 

Two recent studies on a large number of patients have proposed a combination of clinical, biological, and echocardiographic parameters. The first one is the GARFIELD AF tool [107], which derives from a very large multicentric international cohort and incorporates a large number of clinical parameters. Its performance in predicting stroke, death, and bleeding was superior to CHA2DS2-VASc and HAS-BLED in the study, not only in intermediate or high-risk patients but also in low-risk patients. The second one is the CLOTS AF score developed by Sagan et al. [103]. In their cohort of 1000 patients undergoing TEE in patients in AF before cardioversion or ablation, the only factors associated in multivariate analysis with an increased risk of LAA thrombi were creatinine blood level, left ventricular ejection fraction, left atrial volume index, TAPSE, prior stroke, and AF rhythm. The score developed from those parameters showed better performance in predicting LAA thrombi than the CHA2DS2-VASc score.

## 6. Future Directions

### 6.1. Causality of AF in Stroke

Although AF is associated with an increased risk of stroke, a debate remains about whether AF is a causal factor in the occurrence of stroke, or just a marker of risk. Malik et al. [120] used the Bradford Hill criteria to evaluate the causality of AF in the occurrence of stroke or TEs and highlighted that strong data supported the causal hypothesis. One of the missing elements for proving the causal hypothesis is the evidence of a temporal association between an episode of atrial arrythmia and stroke. This lack of temporal association might mean that not all episodes of atrial arrythmia are equal in term of thrombogenicity and thromboembolic risk: the stroke risk associated with an episode of atrial arrythmia might be influenced by not only the characteristics of the episode (the duration of the episode, type of atrial arrythmia, mean cycle length in the LA, or the hour of the episode during the day) but also by all the changes that surround the episode: hemodynamic (heart failure and modification of LA and LAA flow), biological (inflammation, hypercoagulability, and endothelial activation), and clinical (patient daily activity). All those elements might be clues for identifying episodes of AF at high risk of stroke and could improve our capacity to determine the temporal relationship between a high-risk episode of atrial arrythmia and stroke in further studies.

### 6.2. The Need for Improved Risk Stratification

In contemporary medicine, ischemic stroke risk in AF patients is calculated using the CHA2DS2-VASc score. Strong guideline recommendations are tied to this score with respect to the management of thromboembolic risk. OAT use should be considered (class IIa) in males with a score > 1 and in females with a score > 2, and is recommended (class Ia) in males with a score > 2 and in females with a score > 3 [47]. Thanks to the simplicity of the CHA2DS2-VASc score, it is widely used.

Despite the central position that CHA2DS2-VASc has in current clinical practice, its predictive accuracy remains modest, with a C-statistic of 0.578 for prediction of thromboembolism [99,121]. An optimal risk score should perform dynamic risk stratification with precision, and include a complete panel of variables on the one hand and affordability and ease of use on the other hand. Increasing worldwide access to the internet could make the use of a more complex, holistic model feasible through the use of an online risk prediction tool. Such a tool could overcome the need for a simplistic model that, for instance, categorizes continuous variables, hopefully grasping the interplay between factors leading to stroke in patients that have (a predisposition to developing) AF.

The ongoing MAESTRIA study (“Machine Learning and Artificial Intelligence for Early Detection of STroke and Atrial Fibrillation”) aims to further map the substrate for stroke in 600 patients with paroxysmal, persistent, or permanent AF. In this international, observational study, a wealth of information is collected, including imaging and electrocardiographic, omics-based, and hematologic biomarkers, which will be further analyzed using artificial intelligence models. It will be very interesting to see whether these novel technologies will improve the risk stratification for stroke in AF.

### 6.3. A Dynamic Evaluation

Moreover, stroke and systemic embolic risk do not remain the same over time and require dynamic evaluation and frequent update to evaluate the need for anticoagulation treatment during the follow-up of the patient, due to the changes that can occur in the state of the patient in terms of the natural evolution of AF (from paroxysmal to persistent, with an increase in burden), the evolution of atrial cardiomyopathy, or the evolution of comorbidities. Chao et al. [122] showed that in a population of patients with newly diagnosed AF with initial low risk (CHA2DS2-VASc = 0 for men or CHA2DS2-VASc = 1 for women), during the follow-up, the CHA2DS2-VASc score increased each year in 12% of the population. Reevaluation and the prescription of anticoagulants reduced the risk of stroke and was associated with better outcomes.

## 7. Conclusions

Strong evidence supports the causality of AF in the occurrence of a stroke or a thromboembolic event in terms of physiopathology and demographic data. Stroke is the result of a complex and multifactorial process which is supported by both general and intracardiac factors. Thus, numerous risk factors are identifiable, and the performance of the actual risk stratification remains modest. Further studies are needed to create a holistic and dynamic risk stratification which would incorporate all the identified risk factors, in order to reduce the risk of stroke in patients with AF.

## Figures and Tables

**Table 1:** Temporal association between AF and a stroke/TE.

Study	n	Study Design	Main Results
Daoud et al., 2021 [95]	40	TRENDS study subgroup Patients with a stroke/TE + 30 days monitoring on a CIED prior to stroke AF ≥ 5 min with atrial rhythm ≥ 175 bpm	20 patients with AF prior to stroke (9 persistent AF, 11 paroxysmal AF) Only 11 patients (55%) with ≥1 AF episode within 30 days prior to stroke with 6 patients in AF at stroke time Only 6 patients with AF episode after stroke
Brambatti et al., 2014 [35]	51	ASSERT trial subgroup Stroke/TE + 3 months monitoring on a CIED prior to stroke/TE AF ≥ 6 min with atrial rhythm ≥ 190 bpm	18 patients (35%) with ≥1 episode of AF prior to stroke Only 4 patients (8%) with ≥1 episode of AF in the 30 days prior to stroke 1 patient in AF at stroke time Only 8 patients with AF episode after stroke
Turakhia et al., 2015 [97]	187	Healthcare System database Case Crossover Study Stroke/TE + 120 days monitoring on a CIED AF ≥ 5.5 h/day	19% of patients with ≥1 AF episode prior to stroke 16 patients with informative discordant pattern with adjusted OR for ischemic stroke of 5.22 (95% CI: 1.22–47.4) Risk of stroke highest within 5 days following an episode of AF with adjusted OR 17.4 (95% CI: 5.4–73.1), return to baseline after 30 days
Singer et al., 2021 [96]	891	HealthCare database Case crossover study Stroke/TE + 120 days monitoring on CIED AF ≥ 5.5 h/day	24% of patients with ≥1 AF episode prior to stroke 66 patients with informative discordant pattern with OR for ischemic stroke of 3.71 (95% CI: 2.06–6.70) Risk of stroke highest within 5 days following an episode of AF with adjusted OR 5.00 (95% CI: 2.62–9.55) Highest risk of stroke if AF ≥ 23 h on a given day

AF: Atrial Fibrillation; TE: Thromboembolic Event; CIED: Cardiac Implantable Electronic Device; OR: Odds Ratio; CI: Confidence Interval.

## Data Availability

Not applicable.
